# A novel scoring system predicts adjuvant chemolipiodolization benefit for hepatocellular carcinoma patients after hepatectomy

**DOI:** 10.18632/oncotarget.8333

**Published:** 2016-03-24

**Authors:** Li-feng Huang, Xianglei Xing, Dong Wu, Yong Xia, Jun Li, Kui Wang, Zhen-lin Yan, Xu-ying Wan, Le-hua Shi, Tian Yang, Wan Yee Lau, Meng-chao Wu, Feng Shen

**Affiliations:** ^1^ Department of Hepatic Surgery IV, The Eastern Hepatobiliary Surgery Hospital, Second Military Medical University, Shanghai, China; ^2^ Department of Hepatic Surgery I, The Eastern Hepatobiliary Surgery Hospital, Second Military Medical University, Shanghai, China; ^3^ Department of Hepatic Surgery II, The Eastern Hepatobiliary Surgery Hospital, Second Military Medical University, Shanghai, China; ^4^ Department of Clinical Database, The Eastern Hepatobiliary Surgery Hospital, Second Military Medical University, Shanghai, China; ^5^ Faculty of Medicine, The Chinese University of Hong Kong, Shatin, Hong Kong SAR, China

**Keywords:** hepatocellular carcinoma, liver resection, adjuvant chemolipiodolization, prognosis, scoring system

## Abstract

Our aim in this study was to develop a prognostic scoring system with which to identify patients most likely to benefit from adjuvant chemolipiodolization (ACL) after liver resection for hepatocellular carcinoma (HCC). Data from 1150 HCC patients who underwent liver resection between 2002 and 2008 at the Eastern Hepatobiliary Surgery Hospital were used to develop the scoring system. Patients were stratified into prognostic subgroups using the new scoring system, and the outcomes of patients who received ACL and those who did not were compared in each subgroup. Using data from 379 patients operated on between 2008 and 2010 for validation, the scoring system had a concordance index (C-index) of 0.75 for predicting post-resectional overall survival (OS). It optimally stratified patients into three prognostic subgroups with scores of 0–5, 6–9 and ≥ 10, having better, medium and worse survival outcomes, respectively. A difference in OS between ACL and non-ACL patients was only detected in the subgroup with scores ≥ 10 (1-, 3-, and 5-year OS rates: 63.9%, 22.6%, and 9.0% vs. 33.8%, 5.6%, and 2.8%, *p* = 0.001). Our proposed scoring system provides an effective tool for selecting the patients most likely to benefit from ACL.

## INTRODUCTION

Although liver resection remains the first line treatment for patients with hepatocellular carcinoma (HCC), the prognosis is still bad because of high tumor recurrence rate [1]. There is no universally accepted adjuvant procedure for HCC patients after R0 liver resection [2, 3]. Adjuvant transarterial chemoembolization with Lipiodol (adjuvant chemolipiodolization, ACL) is commonly used, but its effectiveness remains controversial [4–6]. Previous studies on this subject varied significantly in study design, inclusion criteria, sample sizes and therapeutic protocols, making comparison difficult [5, 7–9]. However, three recent studies suggest that ACL may benefit patients with high risks of early tumor recurrence, such as those with large tumors, non-encapsulated tumors, and vascular invasion [4, 9, 10].

Dividing surgically treated HCC patients into prognostic subgroups is a challenge because multiple risk factors are involved. Two approaches have been used. The first uses host, surgical, and pathological factors such as serum alpha fetoprotein (AFP), hepatitis activity, tumor diameter, tumor number, and vascular invasion to predict surgical prognosis [11–13]. The obvious down-side of this approach is the unintentional non-inclusion of other important factors with prognostic significance. Another approach utilizes existing clinical staging systems [14, 15]. This method may not properly reflect postoperative prognosis because these systems were not originally built for surgically treated HCC patients. Moreover, the heterogeneity in prognosis of patients within the same stage classified by these systems is obvious [15]. Although specific molecular biomarkers are more accurate predictors, the appropriate laboratory tests are not commonly used clinically [16–18]. A reasonable tool to predict postoperative survival could be used to identify patients who can benefit from adjuvant treatment based on survival risk stratification.

## RESULTS

### Clinicopathologic characteristics

During the study period, 2160 patients underwent liver resection for HCCs in our departments. Based on the pre-defined inclusion criteria, 1529 patients were included. Of these, 1150 formed the primary cohort whose data was used to develop the scoring system; another 379 patients served as the validation cohort. There were no differences in baseline clinicopathologic features between the 2 cohorts (Table 1).

**Table 1:** Clinicopathological characteristics

Variable	Number (percentage)			*P* value
	All patients (*n* = 1529)	Primary cohort (*n* = 1150)	Validation cohort (*n* = 379)	
**Age, years**				
≤ 50	718 (47.0%)	532 (46.3%)	186 (49.1%)	0.34
> 50	811 (53.0%)	618 (53.7%)	193 (50.9%)	
**Gender**				
Male	1311 (85.7%)	995 (86.5%)	316 (83.4%)	0.13
Female	218 (14.3%)	155 (13.5%)	63 (16.6%)	
**HBsAg**				
Positive	1283 (83.9%)	971 (84.4%)	312 (82.3%)	0.33
Negative	246 (16.1%)	179 (15.6%)	67 (17.7%)	
**HBeAg**				
Positive	458 (30.0%)	358 (31.1%)	100 (26.4%)	0.08
Negative	1071 (70.0%)	792 (68.9%)	279 (73.6%)	
**HBcAb**				
Positive	1413 (92.4%)	1060 (92.2%)	353 (93.1%)	0.54
Negative	116 (7.6%)	90 (7.8%)	26 (6.9%)	
**HCVAb**				
Positive	44(2.9%)	34(3.0%)	10(2.6%)	0.75
Negative	1485(97.1%)	1116(97.0%)	369(97.4%)	
**Cirrhosis**				
Yes	832 (54.4%)	617 (53.7%)	215 (56.7%)	0.30
No	697 (45.6%)	533 (46.3%)	164 (43.3%)	
**AFP, ng/mL**				
≤ 400	1023(66.9%)	765(66.5%)	258(68.1%)	0.58
> 400	506(33.1%)	385(33.5)	121(31.9%)	
**PT, seconds**				
≤ 12	801 (52.4%)	607 (52.8%)	194 (51.2%)	0.59
> 12	728 (47.6%)	543 (47.2%)	185 (48.8%)	
**PLT, ×10^9^/L**				
≤ 100	333 (21.8%)	261 (22.7%)	72 (19.0%)	0.13
> 100	1196 (78.2%)	889 (77.3%)	307 (81.0%)	
**ALB, g/L**				
≤ 40	512 (33.5%)	381 (33.1%)	131 (34.6%)	0.61
> 40	1017 (66.5%)	769 (66.9%)	248 (65.4%)	
**ALT, U/L**				
≤ 40	775 (50.7%)	574 (49.9%)	201 (53.0%)	0.29
> 40	754 (49.3%)	576 (50.1%)	178 (47.0%)	
**TBIL, μmol/L**				
≤ 34	1510 (98.8%)	1135 (98.7%)	375 (98.9%)	0.91*
> 34	19 (1.2%)	15 (1.3%)	4 (1.1%)	
**WBC, ×10^9^/L**				
≤ 4	301 (19.7%)	225 (19.6%)	76 (20.1%)	0.84
> 4	1228 (80.3%)	925 (80.4%)	303 (79.9%)	
**Tumor number**				
Single	1286 (84.1%)	963 (83.7%)	323 (85.2%)	0.49
Multiple	243 (15.9%)	187 (16.3%)	56 (14.8%)	
**Tumor diameter, cm**				
≤ 3	410 (26.8%)	292 (25.4%)	118 (31.1%)	0.07
3–5	425 (27.8%)	321 (27.9%)	104 (27.5%)	
> 5	694 (45.4%)	537 (46.7%)	157 (41.4%)	
**MVI**				
Yes	516 (33.7%)	387 (33.7%)	129 (34.0%)	0.89
No	1013 (66.3%)	763 (66.3%)	250 (66.0%)	
**Tumor capsule**				
Complete	834 (54.5%)	641 (55.7%)	193 (50.9%)	0.10
Incomplete	695 (45.5%)	509 (44.3%)	186 (49.1%)	
**Edmondson-Steiner**				
I/II	456 (29.8%)	329 (28.6%)	127 (33.5%)	0.07
III/IV	1073 (70.2%)	821 (71.4%)	252 (66.5%)	
**Blood transfusion**				
Yes	178 (11.6%)	139 (12.1%)	39 (10.3%)	0.34
No	1351 (88.4%)	1011 (87.9%)	340 (89.7%)	
**Surgical margin, cm**				
≤ 1	995 (65.1%)	736 (64.0%)	259 (68.3%)	0.12
> 1	534 (34.9%)	414 (36.0%)	120 (31.7%)	
**Hepatectomy**				
Anatomical	692 (45.3%)	536 (46.6%)	156 (41.2%)	0.07
Non-anatomical	837 (54.7%)	614 (53.4%)	223 (58.8%)	
**ACL**				
Yes	523 (34.2%)	393 (34.2%)	130 (34.3%)	0.96
No	1006 (65.8%)	757 (65.8%)	249 (65.7%)	

* Continuity Correction test.

Abbreviation: HBsAg, hepatitis B surface antigen; HBeAg, hepatitis B e antigen; HBcAb, hepatitis B core antibody; HCVAb, hepatitis C virus antibody; AFP, alpha fetoprotein; PT, prothrombin time; PLT, platelets; ALB, albumin; ALT, alanine transaminase; TBIL, total bilirubin; WBC, white blood cell; MVI, microvascular invasion; ACL, adjuvant chemolipiodolization.

The clinicopathologic characteristics of the ACL and non-ACL patients in these 2 cohorts were compared (Table 2). In the primary cohort, fewer patients in the ACL group received intraoperative blood transfusion (9.4% vs. 13.5%, *p* = 0.04). In the validation cohort, more patients in the ACL group had HBcAb positivity (97.7% vs. 90.8%, *p* = 0.01), and had poorly differentiated tumors (grade III/IV, 73.8% vs. 62.7%, *p* = 0.03).

**Table 2:** Clinicopathologic characteristics of patients treated with or without ACL in the primary and validation cohorts

Variable	Primary cohort			Validation cohort		
	Non-ACL	ACL	*P* value	Non-ACL	ACL	*P* value
	(*n* = 757)	(*n* = 393)		(*n* = 249)	(*n* = 130)	
**Age, years**						
≤ 50	341 (45.0%)	191 (48.6%)	0.25	121 (48.6%)	65 (50.0%)	0.80
> 50	416 (55.0%)	202 (51.4%)		128 (51.4%)	65 (50.0%)	
**Gender**						
Male	652 (86.1%)	343 (87.3%)	0.59	207 (83.1%)	109(83.8%)	0.86
Female	105 (13.9%)	50 (12.7%)		42 (16.9%)	21(16.2%)	
**HBsAg**						
Positive	631 (83.4%)	340 (86.5%)	0.16	201 (80.7%)	111 (85.4%)	0.26
Negative	126 (16.6%)	53 (13.5%)		48 (19.3%)	19 (14.6%)	
**HBeAg**						
Positive	236 (31.2%)	122 (31.0%)	0.96	69 (27.7%)	31 (23.8%)	0.42
Negative	521 (68.8%)	271 (69.0%)		180 (72.3%)	99 (76.2%)	
**HBcAb**						
Positive	701 (92.6%)	359 (91.3%)	0.45	226 (90.8%)	127 (97.7%)	0.01
Negative	56 (7.4%)	34 (8.7%)		23 (9.2%)	3 (2.3%)	
**HCVAb**						
Positive	24 (3.2%)	10 (2.5%)	0.55	8 (3.2%)	2 (1.5%)	0.33
Negative	733 (96.8%)	383 (97.5%)		241 (96.8%)	128 (98.5%)	
**Cirrhosis**						
Yes	396 (52.3%)	221 (56.2%)	0.21	143 (57.4%)	72 (55.4%)	0.70
No	361 (47.7%)	172 (43.8%)		106 (42.6%)	58 (44.6%)	
**AFP, ng/mL**						
≤ 400	496 (65.5%)	269 (68.4%)	0.32	168 (67.5%)	90 (69.2%)	0.73
> 400	261 (34.5%)	124 (31.6%)		81 (32.5%)	40 (30.8%)	
**PT, second**						
≤ 12	404 (53.4%)	203 (51.7%)	0.58	126 (50.6%)	68 (52.3%)	0.75
> 12	353 (46.6%)	190 (48.3%)		123 (49.4%)	62 (47.7%)	
**PLT, ×10^9^/L**						
≤ 100	174 (23.0%)	87 (22.1%)	0.75	49(19.7%)	23 (17.7%)	0.64
> 100	583 (77.0%)	306 (77.9%)		200(80.3%)	107 (82.3%)	
**ALB, g/L**						
≤ 40	259 (34.2%)	122 (31.0%)	0.28	83 (33.3%)	48 (36.9%)	0.49
> 40	498 (65.8%)	271 (69.0%)		166 (66.7%)	82 (63.1%)	
**ALT, U/L**						
≤ 40	379 (50.1%)	195 (49.6%)	0.89	135(54.2%)	66 (50.8%)	0.52
> 40	378 (49.9%)	198 (50.4%)		114(45.8%)	64 (49.2%)	
**TBIL, μmol/L**						
≤ 34	747 (98.7%)	388 (98.7%)	0.95	246(98.8%)	129 (99.2%)	0.99*
> 34	10 (1.3%)	5 (1.3%)		3 (1.2%)	1 (0.8%)	
**WBC, ×10^9^/L**						
≤ 4	152 (20.1%)	73 (18.6%)	0.54	50 (20.1%)	26 (20.0%)	0.98
> 4	605 (79.9%)	320 (81.4%)		199 (79.9%)	104 (80.0%)	
**Tumor number**						
Single	626 (82.7%)	337 (85.8%)	0.18	216 (86.7%)	107 (82.3%)	0.25
Multiple	131 (17.3%)	56 (14.2%)		33 (13.3%)	23 (17.7%)	
**Tumor diameter, cm**						
≤ 3	181 (23.9%)	111 (28.2%)	0.27	84 (33.7%)	34 (26.2%)	0.17
3–5	217 (28.7%)	104 (26.5%)		70 (28.1%)	34 (26.2%)	
> 5	359 (47.4%)	178 (45.3%)		95 (38.2%)	62 (47.6%)	
**MVI**						
Presence	250 (33.0%)	137 (34.9%)	0.53	83 (33.3%)	46 (35.4%)	0.69
Absence	507 (67.0%)	256 (65.1%)		166 (66.7%)	84 (64.6%)	
**Tumor capsule**						
Complete	425 (56.1%)	216 (55.0%)	0.70	129 (51.8%)	64 (49.2%)	0.63
Incomplete	332 (43.9%)	177 (45.0%)		120 (48.2%)	66 (50.8%)	
**Edmondson-Steiner**						
I/II	213 (28.1%)	116 (29.5%)	0.62	93 (37.3%)	34 (26.2%)	0.03
III/IV	544 (71.9%)	277 (70.5%)		156 (62.7%)	96 (73.8%)	
**Blood transfusion**						
Yes	102 (13.5%)	37 (9.4%)	0.04	22 (8.8%)	17 (13.1%)	0.20
No	655 (86.5%)	356 (90.6%)		227 (91.2%)	113 (86.9%)	
**Surgical margin, cm**						
≤ 1	486 (64.2%)	250 (63.6%)	0.84	165 (66.3%)	94 (72.3%)	0.23
> 1	271 (35.8%)	143 (36.4%)		84 (33.7%)	36 (27.7%)	
**Hepatectomy**						
Anatomical	364 (48.1%)	172 (43.8%)	0.16	108 (43.4%)	48 (36.9%)	0.23
Non-anatomical	393 (51.9%)	221 (56.2%)		141 (56.6%)	82 (63.1%)	

* ontinuity Correction test.

Abbreviation: HBsAg, hepatitis B surface antigen; HBeAg, hepatitis B e antigen; HBcAb, hepatitis B core antibody; HCVAb, hepatitis C virus antibody; AFP, alpha fetoprotein; PT, prothrombin time; PLT, platelets; ALB, albumin; ALT, alanine transaminase; TBIL, total bilirubin; WBC, white blood cell; MVI, microvascular invasion; ACL, adjuvant chemolipiodolization.

### Tumor recurrence, OS and independent risk factors in the primary cohort

The follow-up was censored on June 30, 2013. The median follow-up period was 34.6 months (range, 1.4 to 107.8). The median overall survival (OS) was 23.1 months (range, 1.2 to 106.3), and the 1-, 3-, and 5-year OS rates were 88.5%, 68.1%, and 45.3%, respectively. The median time to recurrence (TTR) was 18.5 months (range, 1.1 to 106.3), and the 1-, 3-, and 5-year recurrence rates were 32.9%, 56.3%, and 66.6%, respectively.

The results of the univariable analysis of OS and recurrence are shown in Supplementary Table 1. Multivariable analysis identified tumor diameter (3–5 cm: hazard ratio [HR] 1.89, 95% confidence interval [CI], 1.29–2.75; > 5 cm: 4.27, 3.05–5.97), multiple tumors (1.42, 1.12–1.81), presence of microvascular invasion (MVI) (2.45, 1.98–3.02), incomplete tumor capsule (1.82, 1.47–2.25), and surgical margin ≤ 1.0 cm (1.38, 1.11–1.72) as independent risk factors for OS. The independent risk factors for recurrence were similar to those for OS, with HBeAg positivity as an additional risk factor (1.22, 1.02–1.46) (Table 3).

**Table 3:** Multivariable analysis for tumor recurrence and OS in the primary cohort

Variable*	Tumor recurrence		OS	
	*P*	HR (95.0% CI)	*P*	HR (95.0% CI)
**HBsAg**				
Positive vs. Negative	0.34	1.12 (0.88–1.43)	0.06	1.33 (0.99–1.78)
**HBeAg**				
Positive vs. Negative	0.03	1.22 (1.02–1.46)		
**AFP, ng/mL**				
> 400 vs. ≤ 400	0.65	1.04 (0.87–1.24)	0.10	0.83 (0.67–1.04)
**ALT, U/L**				
> 40 vs. ≤ 40	0.09	1.15 (0.98–1.36)		
**Tumor number**				
Multiple vs. Single	0.001	1.39 (1.14–1.70)	0.005	1.42 (1.12–1.81)
**Tumor diameter, cm**				
3–5 vs. ≤ 3	0.001	1.57 (1.21–2.03)	0.001	1.89 (1.29–2.75)
> 5 vs. 3–5	< 0.001	2.65 (2.10–3.34)	< 0.001	4.27 (3.05–5.97)
**MVI**				
Presence vs. Absence	< 0.001	2.70 (2.28–3.19)	< 0.001	2.45 (1.98–3.02)
**Tumor capsule**				
Incomplete vs. Complete	0.007	1.25 (1.06–1.48)	< 0.001	1.82 (1.47–2.25)
**Edmondson–Steiner**				
III/IV vs. I/II	0.79	1.03 (0.84–1.25)	0.60	1.07 (0.83–1.38)
**Blood transfusion**				
Yes vs. No	0.58	0.93 (0.73–1.19)		
**Surgical margin, cm**				
≤ 1 vs. > 1	0.01	1.24 (1.05–1.47)	0.004	1.38 (1.11–1.72)

* All the variables listed in Supplementary table 1 were used for the univariable analysis and only the significant factors were subjected to the multivariable analysis.

### Development of the scoring system for OS prediction in the primary cohort

The regression coefficients of the 5 independent risk factors for OS were 0.635 for 3–5 cm and 1.451 for > 5 cm tumor diameters, 0.352 for multiple tumors, 0.895 for presence of MVI, 0.600 for incomplete tumor capsule, 0.324 for ≤ 1.0 cm surgical margin. The weights of these predictors were 2 for tumor diameter 3–5 cm (0.635/0.324), 4 for tumor diameter > 5 cm (1.451/0.324), 1 for multiple tumors (0.352/0.324), 3 for MVI presence (0.895/0.324), 2 for incomplete tumor capsule (0.600/0.324), and 1 for surgical margin ≤ 1.0 cm (0.324/0.324), respectively.

Using the weighted sum method, a scoring system predicting OS was formulated: MVI (presence = 3, absence = 0) + capsule (incomplete = 2, complete = 0) + tumor diameter (> 5 cm = 4, 3–5 cm = 2, ≤ 3 cm = 0) + tumor number (multiple = 1, single = 0) + surgical margin (≤ 1 cm = 1, > 1 cm = 0) (Table 4).

**Table 4:** Prognostic risk scores

Variable	Score
**MVI**	
Presence	3
Absence	0
**Tumor capsule**	
Incomplete	2
Complete	0
**Tumor diameter, cm**	
> 5	4
3–5	2
≤ 3	0
**Tumor number**	
Multiple	1
Single	0
**Surgical margin, cm**	
≤ 1	1
> 1	0
***Prognostic subgroups***	***Total score***
Better	0–5
Medium	6–9
Worse	10–11

**Figure 1:**
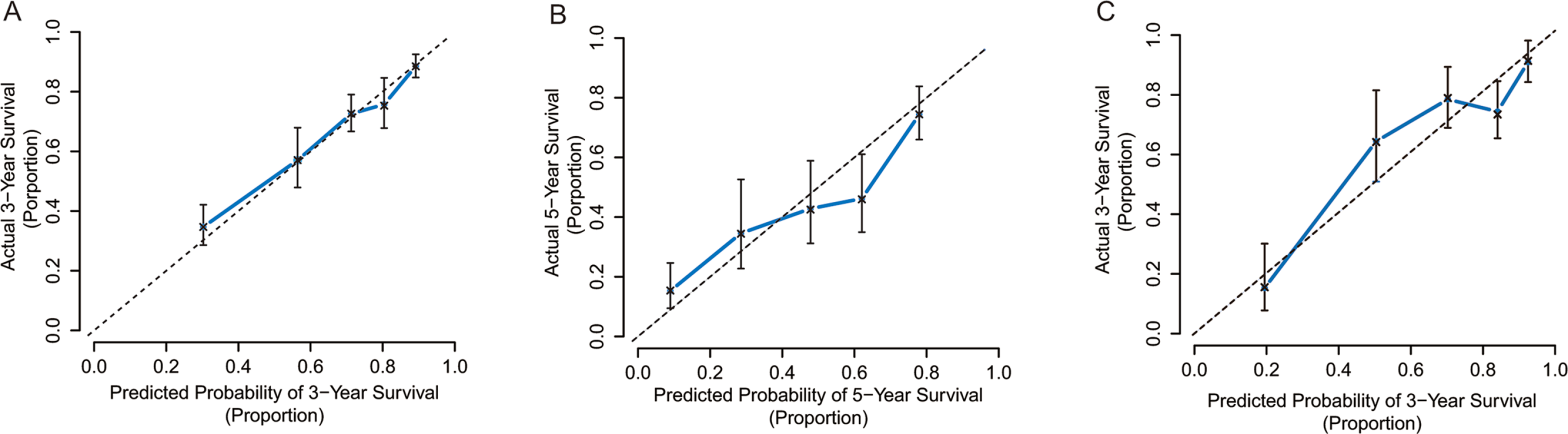
Calibration curve analysis for OS by the scoring system (**A**) 3-year after liver resection in the primary cohort; (**B**) 5-year after liver resection in the primary cohort; (**C**) 3-year after liver resection in the validation cohort.

The C-index of the scoring system for predicting OS was 0.75 (95% CI, 0.72 to 0.78). Our calibration curves show strong correlation between the prediction by the scoring system and actual observation (Figure 1A, 1B).

### Prognostic subgroups stratified by the scoring system in the primary cohort

**Figure 2:**
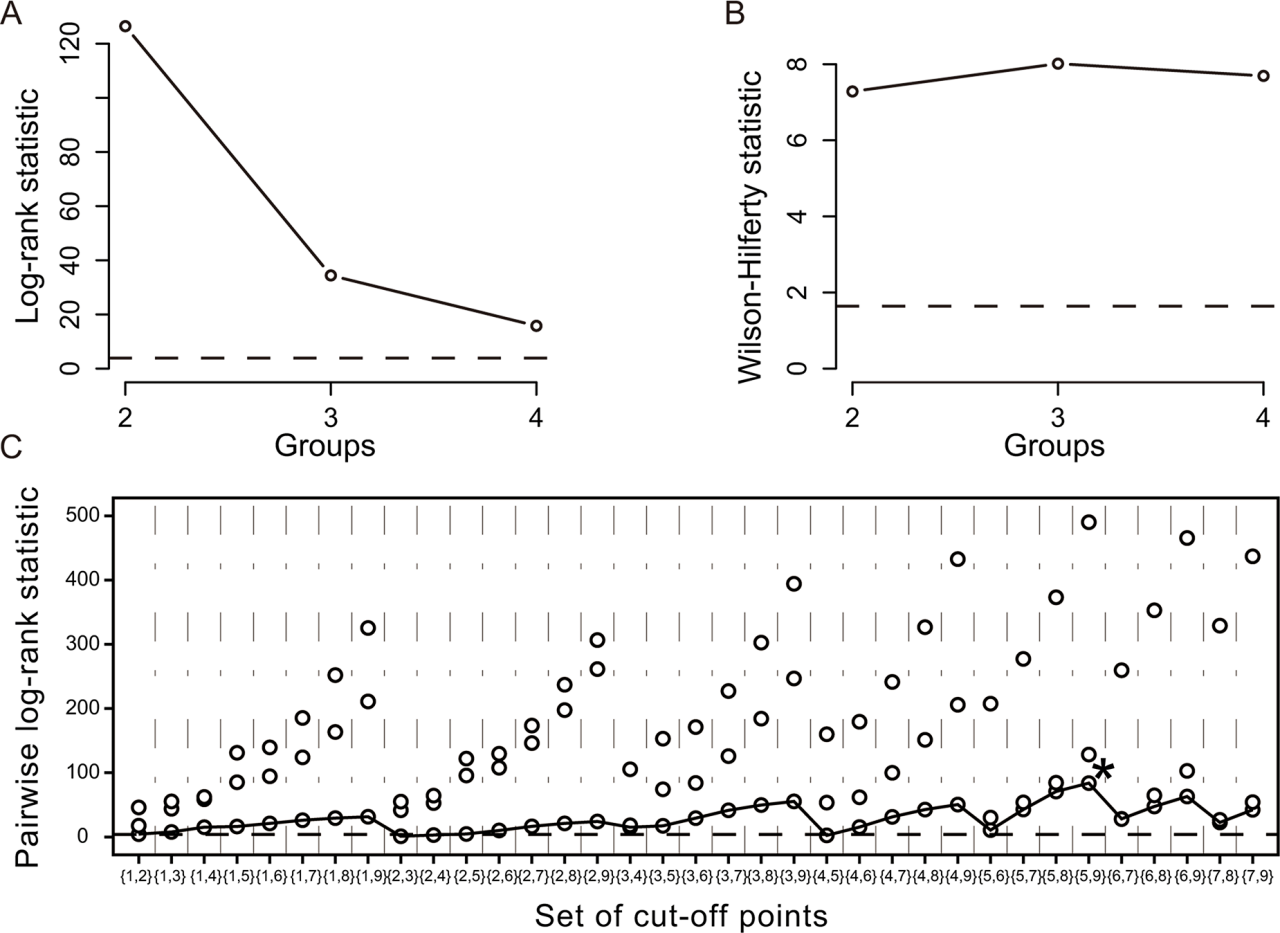
Stratification of patients by the K-adaptive partitioning statistical algorithm in the primary cohort (**A**) Log-rank statistic for the number of groups (K = 2, 3, 4) by cross-validation; (**B**) Wilson-Hilferty transformation of the log-rank statistics; (**C**) Pairwise log-rank statistics when the number of groups (K) is 3. (The star symbol indicates the best set of cut-off points).

**Figure 3:**
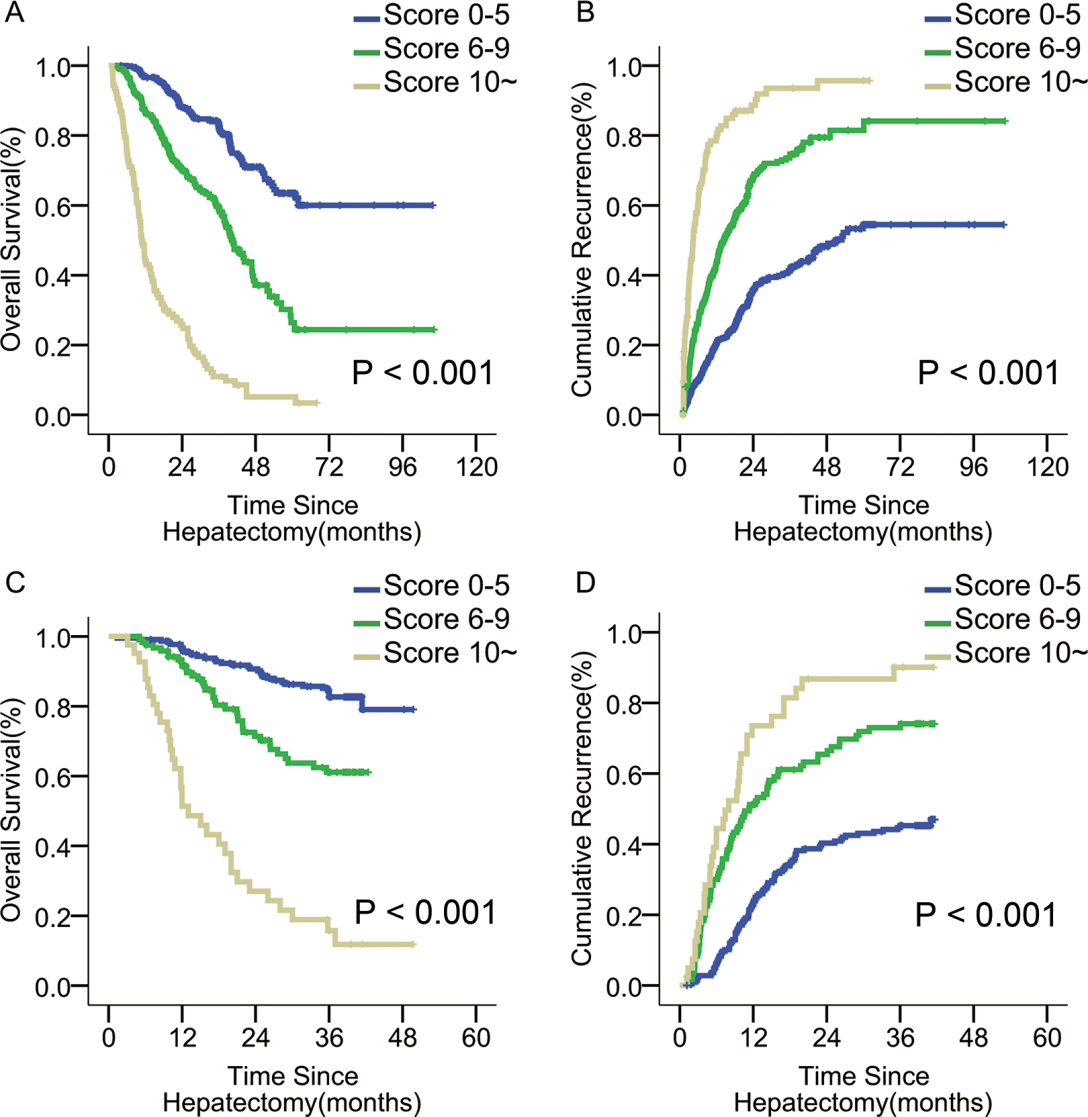
OS and tumor recurrence after liver resection for patients with different risk scores in the primary and validation cohorts (**A**, **B**) OS and tumor recurrence in the primary cohort; (*p* < 0.001 for both) (**C**, **D**) OS and tumor recurrence in the validation cohort. (*p* < 0.001 for both).

Using K-adaptive partitioning, patients were stratified into 3 distinct incremental prognostic subgroups with two optimal cut-off scores of 5 and 9 (Figure 2). These 3 patient subgroups with scores of ≤ 5 (*n* = 680), 6–9 (*n* = 363), and ≥10 (*n* = 107) had different 1-, 3-, and 5-year OS rates (96.6%, 82.9%, and 63.5% vs. 86.5%, 59.2%, and 26.4% vs. 43.9%, 11.0%, and 5.1%, *p* < 0.001), and corresponding recurrence rates (20.4%, 41.2%, and 54.5% vs. 43.7%, 74.6%, and 81.5% vs. 79.5%, 93.5%, and 95.7%, *p* < 0.001) (Figure 3A, 3B).

The Barcelona Clinic Liver Cancer (BCLC) and the seventh edition of tumor-node-metastasis (7th TNM) systems also stratify patients into several subgroups [19, 20], achieving C-indexes of 0.65 and 0.58 for OS prediction, respectively, which were lower than that of our scoring system (0.75, both *p* < 0.001).

### Impact of adjuvant chemolipiodolization on the three prognostic subgroups in the primary cohort

447 of the 680 patients in the subgroup with a score of ≤ 5, 124 of the 363 in the 6–9 subgroup, and 36 of the 107 patients scoring ≥ 10 received ACL.

**Figure 4:**
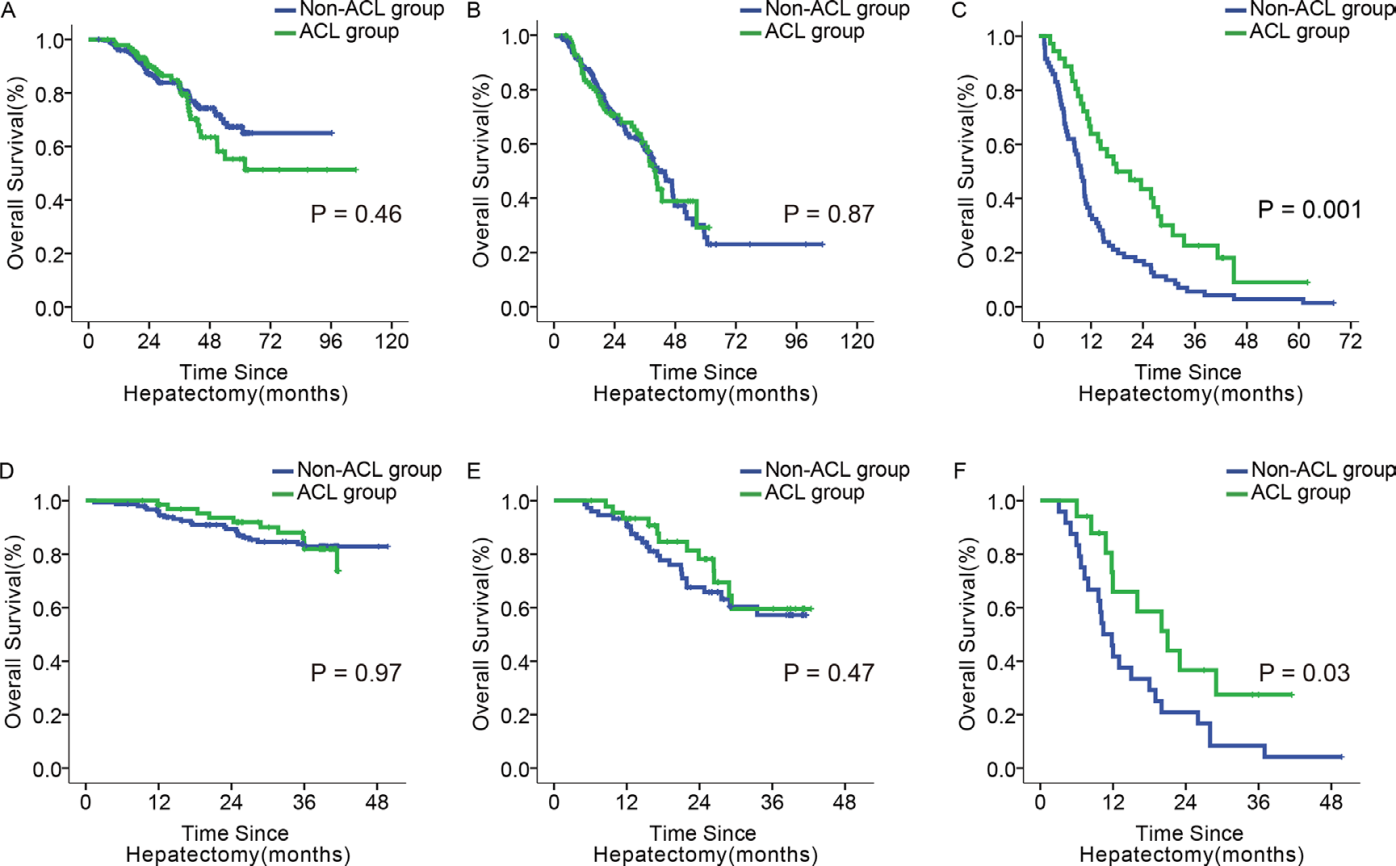
Impact of ACL on OS in patients with different scores (**A–C**) OS of ACL and non-ACL patients with the score of 0–5, 6–9, and ≥ 10, in the primary cohort; (**D–F**): OS of ACL and non-ACL patients with the score of 0–5, 6–9, and ≥ 10, in the validation cohort.

The 1-, 3-, and 5-year OS rates were indistinguishable between ACL and non-ACL patients with scores of ≤ 5 and 6–9 (*p* = 0.46, 0.87; Figure 4A, 4B). However, in the subgroup with a score of ≥ 10, the OS rates were higher for the ACL patients at all time points (63.9%, 22.6%, and 9.0% vs. 33.8%, 5.6%, and 2.8%, *p* = 0.001; Figure 4C).

**Figure 5:**
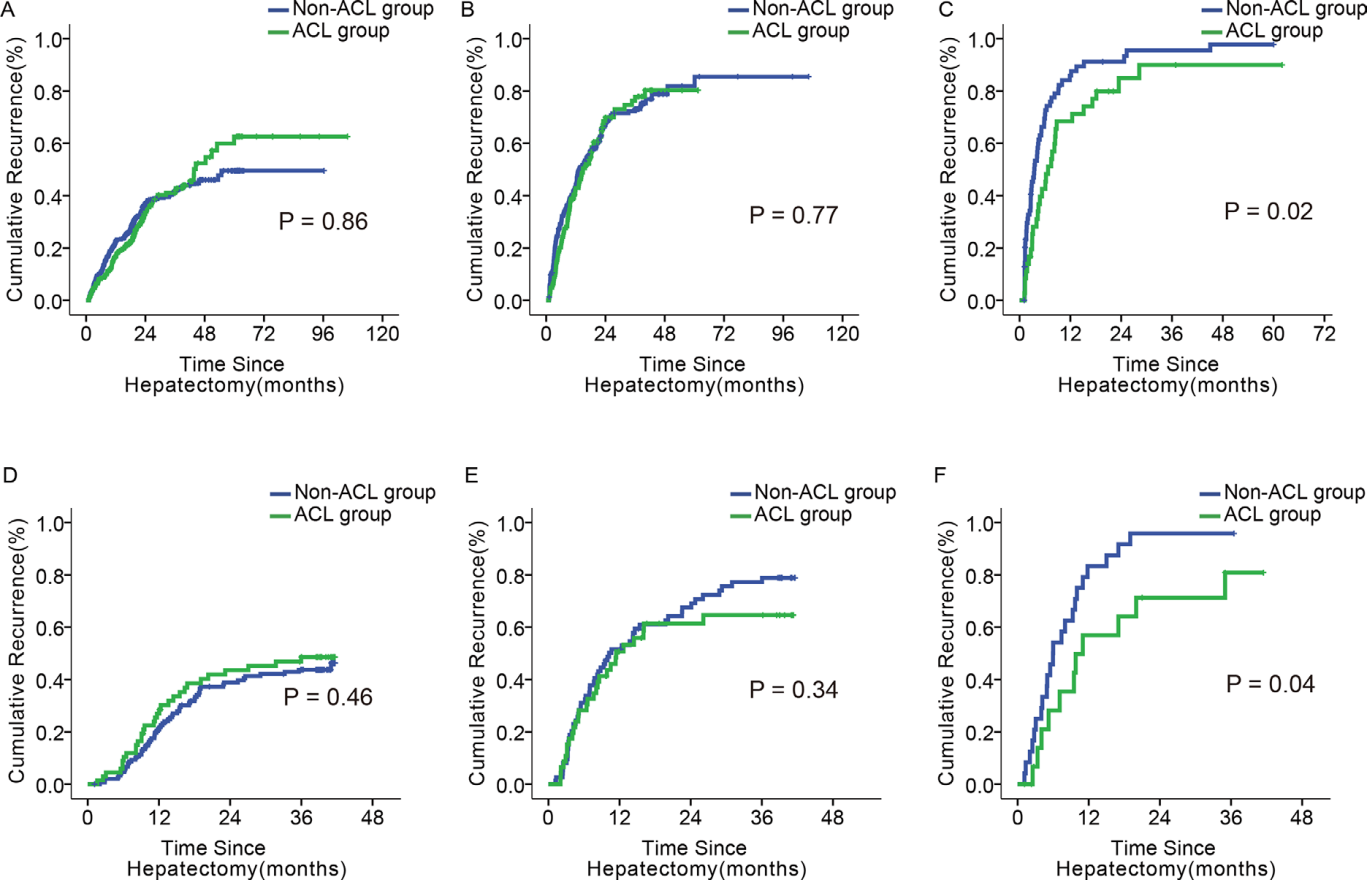
Impact of ACL on tumor recurrence in patients with different scores (**A–C**) Tumor recurrence of ACL and non–ACL patients with the score of 0–5, 6–9, and ≥ 10, in the primary cohort; (**D–F**) Tumor recurrence of and non–ACL patients with the score of 0–5, 6–9, and ≥10, in the validation cohort.

In the subgroup with a score of ≥ 10, the ACL patients had lower 1-, 3-, and 5- year recurrence rates than the non-ACL patients (68.4%, 89.9%, and 89.9% vs. 85.9%, 95.6%, and 97.8%, *p* = 0.02; Figure 5C). Such differences were not identified in the other two subgroups (*p* = 0.86, 0.77; Figure 5A, 5B). There was no difference in OS or recurrence between ACL and non-ACL patients in any of the subgroups classified by the BCLC or 7th TNM systems (Supplementary Figures 2–5).

### Validation

In the validation cohort, the median follow-up period was 36.2 months (range, 2.6 to 49.8). The 1- and 3- year OS rates were 90.3% and 68.6%; and 1- and 3- year recurrence rates were 37.1% and 58.7%, respectively.

By stratification with our scoring system, the ACL patients from the 3 subgroups with scores ≤ 5 (*n* = 218), 6–9 (*n* = 120), and ≥10 (*n* = 41) had better 1- and 3- year OS rates (96.7% and 82.7% vs. 91.5% and 58.3% vs. 51.3% and 15.2%, *p* < 0.001; Figure 3C), and corresponding recurrence rates (22.9% and 45.2% vs. 51.2% and 73.0% vs. 73.5% and 90.1%, *p* < 0.001; Figure 3D) than their non-ACL counterparts. The C-index of the scoring system in predicting OS rate was 0.76, higher than those of the BCLC and 7th TNM systems (0.67 and 0.58, both *p* < 0.001). The calibration curve demonstrates strong correlation between our scores and clinical outcomes (Figure 1C).

67 of 218 patients in the ≤ 5 score subgroup, 46 of 120 in the 6–9 score subgroup, and 17 of 41 patients in the ≥ 10 score subgroup received ACL. Only the ≥ 10 score subgroup had differences between ACL and non-ACL patients in 1- and 3- year OS and recurrence rates (65.9% and 27.5% vs. 41.7% and 8.3%, *p* = 0.03; 56.9% and 80.9% vs. 83.3% and 95.8%, *p* = 0.04; Figure 4F, Figure 5F). These differences were not found in the other two subgroups (Figure 4D, 4E; Figure 5D, 5E). Similarly, there was no significant difference in OS or recurrence between ACL and non-ACL patients in each subgroup stratified with BCLC and 7th TNM systems (Supplementary Figures 2–5).

## DISCUSSION

An accurate method to predict survival outcomes in HCC patients after hepatectomy is still lacking [14, 15]. In this study, we developed a scoring system that optimally predicts post-resectional OS, based on independent predictors of OS including tumor diameter, number, capsule status, presence of MVI, and surgical resection margin. This scoring system, with a C-index of 0.75, possesses good discriminative ability for post-resectional OS. Using this system, patients were stratified into 3 distinct incremental prognostic subgroups. These 3 subgroups, with scores of 0–5, 6–9, and ≥ 10, had better, medium, and worse survival outcomes after liver resection.

ACL is commonly used as an adjuvant therapy after liver resection for HCC [4, 9]. Lipiodol, as an embolic agent in chemoembolization, is a good chemotherapy drug carrier and causes less damage to the liver remnant after liver resection than other embolic materials [21, 22]. However, the adjuvant role of ACL remained controversial in previous studies [4–9, 23, 24]. Although some authors reported ACL improved survival outcomes after liver resection for HCC [4, 23], others showed ACL decreased early tumor recurrence, but failed to prolong long-term survival [6, 7]. One study even suggested that ACL worsened overall survival [24]. These conflicting results may have resulted from differences in study design, patient selection, and sample size. Although ACL may provide survival benefit for patients with high risks of tumor recurrence [5, 6, 9], these patients have not been defined.

We proposed this scoring system to identify patients who would benefit from ACL. When comparing the effectiveness of ACL in 3 subgroups stratified with the scoring system, we found and then confirmed that only those with a score of ≥10, predicted to have the worst survival outcomes, benefited from ACL. Therefore, this scoring system can be used to select the best candidates for ACL after R0 liver resection for HCC.

Although the BCLC and 7th TNM staging systems also divide patients into prognostic subgroups, patients who were treated with ACL did not have any difference from those who did not in OS or recurrence in any of these subgroups. These conventional systems were not built for surgically treated HCC patients; they only recommend patients with early stage tumors for liver resection [19, 20].

In this study, patients with a score ≥ 10 accounted for 9.3% (*n* = 107) and 10.8% (*n* = 41) of the patients in the primary and the validation cohorts, respectively. ACL failed to improve the long-term prognosis of the majority of patients. Our data demonstrates that ACL should be administered carefully and is suitable only for patients with high risk of early tumor recurrence. Inappropriate use of this procedure does not improve surgical efficacy and can lead to liver damage.

Patients with gross portal/hepatic vein invasion such as portal vein tumor thrombus (PVTT) were not included into analysis. The effectiveness of liver resection for these patients is still controversial [25–28] and current studies and guidelines do not support its use [25, 26]. In addition, for these patients who underwent liver resection, ACL was more commonly used and found to be effective [5, 10, 23]. The controversy over ACL was mainly over patients with HCC without gross vascular invasion after a “curative” liver resection.

Our study had limitations: 1) this was a single institutional study; 2) our proposed scoring system is not suitable for patients with major portal/hepatic vein invasion; 3) due it being retrospective study, there might be potential biases affecting the prognostic comparison; 4) as the majority of our patients had HBsAg (83.9%) positivity, our results might not be suitable for patients with HCC etiologies other than HBV infection; 5) The liver functional reserve and general performance of the patient should also be considered in selecting ACL.

In conclusion, this proposed scoring system optimally predicted prognosis of patients who underwent R0 liver resection for HCC, and it was useful in the selection of patients most likely to benefit from ACL.

## MATERIALS AND METHODS

### Study design and data collection

The study was approved by the Institutional Ethics Committee of the Eastern Hepatobiliary Surgery Hospital (EHBH). Informed consent to use their data in research was obtained from all patients before surgery.

Between September 2002 and May 2010, data from 2160 consecutive patients who underwent partial hepatectomy for pathologically proven HCCs at the EHBH were prospectively collected. Patients who received an R0 liver resection were enrolled. An R0 resection was defined as complete removal of macroscopic nodule(s) with a microscopic tumor free resection margin [29]. Patients who had a history of other malignancies, received preoperative anti-cancer therapy, had major portal/hepatic vein tumor invasion and extrahepatic metastasis, died within postoperative 30 days of operation, had incomplete data, or were lost before follow-up were excluded. Based on these criteria, 631 patients were excluded (Supplementary Figure 1) and the remaining 1529 were included. Of these, patients who were operated on from September 2002 to September 2008 formed the primary cohort, and those operated on from October 2008 to May 2010 served as the validation cohort.

Before operation, all patients received routine serology examinations including hepatitis B and C immunology, liver function test, AFP, platelet count (PLT) and prothrombin time (PT). Chest radiograph, abdominal ultrasound, and contrast-enhanced computed tomography (CT) scan and/or magnetic resonance imaging (MRI) of the abdomen were routinely carried out. A preoperative clinical diagnosis of HCC was based on the criteria of the American Association for the Study of Liver Diseases (AASLD) [30].

Liver resection was considered if all tumor nodules detected by preoperative imaging studies were technically resectable without compromising the patient's liver function. Intra-operative ultrasound was routinely used. The type of hepatectomy was selected based on tumor distribution, degree of cirrhosis, and estimated volume of future liver remnant by CT/MRI volumetry [31, 32]. Histopathologic study of surgical specimens was routinely carried out and tumor cell differentiation was determined according to the Edmondson-Steiner grade.

### Adjuvant chemolipiodolization

After operation, patients received similar routine treatment except for the use of ACL. ACL was performed 4 to 8 weeks after the operation on selected patients based on: (1) patients had a WHO performance status of 0–1, a Child-Pugh class A or well B of liver function, a normal kidney function, a white blood cell count of ≥ 3.0×10^9^/L, and a platelet count of ≥ 50 × 10^9^/L. (2) patients with HCC presenting some aggressive pathological features such as multiple tumors, large tumor size, and MVI presence. For patients who did not have these features, ACL was not recommended. (3) a detailed discussion on the pros and cons of ACL between the operating surgeons and the patients.

A vascular catheter was inserted through the femoral artery using the Seldinger technique [33]. ACL regimens consisted of 5-fluorouracil (5-FU, 500 mg/m^2^, Xudong Haipu Pharmaceutical, Shanghai, China), mitomycin C (MMC, 10 mg/m^2^, HiSun Pharmaceutical, Zhejiang, China), cisplatin (DDP, 40 mg/m^2^, Nanjing Pharmaceutical, Nanjing, China) and 5–8 ml of Lipiodol Ultra-Fluide (Guerbet Laboratories, Aulnay-Sous-Bios, France). Patients received ACL once if there was no intrahepatic tumor staining on hepatic arterial angiography prior to chemoembolization. For patients with detected intrahepatic tumor staining, a super-selective vascular catheter was placed into the feeding vessel supplying the tumor, and chemotherapeutic drugs and Lipiodol were delivered and adjusted individually. These patients were included in the ACL group in our analysis.

### Follow-up and endpoints

Patients were followed-up once every 2 months for 2 years and once every 3 to 6 months thereafter. Contrast-enhanced CT scan and/or MRI of the abdomen, and chest radiography or non-contrast CT were performed once every 6 months or earlier if tumor recurrence was suspected. The diagnosis and management of tumor recurrence were similar to those reported previously [34].

In particular, patients who had early tumor recurrence identified by hepatic arterial angiography prior to chemoembolization were investigated with CT scan or MRI of the abdomen one month after chemoembolizaion to evaluate the retention of Lipiodol in the tumors. Sequential treatments using additional TACE, ablation, sorafineb, or conservative treatment were determined based on the effectiveness of the previous chemoembolization, tumor stage, morphology of recurrent tumor and liver function [6, 9].

The endpoints of this study were OS and TTR. OS was defined as the interval between the date of operation and the date of patient's death or last follow-up, while TTR was calculated from the date of surgery to the date when tumor recurrence was diagnosed.

### Statistical analysis

Statistical analyses were performed using R version 3.0.1 and SPSS 15.0 for Windows (SPSS, Chicago, IL). Categorical variables were grouped based on the clinical findings before modeling. The results were compared using the χ^2^ test or Fisher's exact test.

A prognostic scoring system was formulated as previously described [35, 36]. Briefly, it was constructed using the weighted sum method based on independent risk factors of OS. The weights were taken as the corresponding estimated coefficients in a Cox regression analysis after being division the smallest coefficient and rounding to the nearest integer. The predictive accuracy and discriminative ability of the scoring system were determined by concordance index (C-index) and assessed by comparing score-predicted versus observed Kaplan-Meier estimates of survival probability [37]. Bootstraps with 2,000 resamples were used for these activities.

Patients were stratified into prognostic subgroups by comparing differences in survival using the K-adaptive partitioning statistical algorithm [38]. Survival and cumulative recurrence curves of each subgroup were estimated using the Kaplan-Meier method and log-rank test. A *p* < 0.05 was considered statistically significant.

## SUPPLEMENTARY MATERIALS TABLES AND FIGURES


