# Supplementary material for: A novel scoring system predicts adjuvant chemolipiodolization benefit for hepatocellular carcinoma patients after hepatectomy
Source: Oncotarget. 2016 Mar 24;7(18):25493–506. doi: 10.18632/oncotarget.8333 (PMC5041920; doi:10.18632/oncotarget.8333)
Supplement: Supplementary file 1 [file oncotarget-07-25493-s001.pdf]

A novel scoring system predicts adjuvant chemolipiodolization benefit for hepatocellular carcinoma patients after hepatectomy

Supplementary Materials

Supplementary Table S1: Univariable analysis of tumor recurrence and OS in the primary cohort

Variable	Tumor recurrence		OS	
	P	HR (95.0%CI)	*P*	HR (95.0% CI)
**Age, years**				
> 50 vs. ≤ 50	0.50	0.95 (0.81–1.11)	0.09	0.84 (0.69–1.03)
**Gender**				
Male vs. Female	0.13	1.21 (0.95–1.55)	0.40	1.14 (0.84–1.57)
**HBsAg,**				
Positive vs. Negative	0.02	1.31 (1.04–1.64)	0.04	1.36 (1.02–1.82)
**HBeAg**				
Positive vs. Negative	0.02	1.23 (1.04–1.45)	0.07	1.22 (0.98–1.51)
**HBcAb**				
Positive vs. Negative	0.10	1.29 (0.95–1.76)	0.23	1.26 (0.86–1.86)
**HCVAb**				
Positive vs. Negative	0.46	0.83 (0.51–1.37)	0.10	0.51 (0.23–1.13)
**Cirrhosis**				
Positive vs. Negative	0.78	1.02 (0.87–1.20)	0.93	1.01 (0.82–1.24)
**AFP, ng/mL**				
> 400 vs. ≤ 400	< 0.001	1.39 (1.18–1.64)	0.02	1.30 (1.05–1.60)
**PT, second**				
> 12 vs. ≤ 12	0.11	1.14 (0.97–1.34)	0.91	1.01 (0.83–1.24)
**PLT, ×10^9/L**				
>100 vs. ≤ 100	0.46	1.08 (0.89–1.30)	0.12	1.22 (0.95–1.56)
**ALB, g/L**				
> 40 vs. ≤ 40	0.27	0.91 (0.77–1.07)	0.70	1.04 (0.84–1.30)
**ALT, U/L**				
>40 vs. ≤40	0.004	1.26 (1.08–1.48)	0.32	1.11 (0.91–1.36)
**TBIL, μmol/L**				
≤ 34 vs. > 34	0.70	1.15 (0.57–2.30)	0.70	0.83 (0.31–2.21)
**WBC, ×10^9/L**				
> 4 vs. ≤ 4	0.26	1.12(0.92–1.38)	0.10	1.25(0.96–1.63)
**Tumor number**				
Multiple vs. Single	< 0.001	1.94(1.60–2.35)	< 0.001	2.06 (1.63–2.60)
**Tumor diameter, cm**				
3–5 vs. ≤ 3	< 0.001	1.60 (1.24–2.06)	< 0.001	1.96 (1.35–2.85)
> 5 vs. 3–5	< 0.001	2.86 (2.28–3.57)	< 0.001	4.50 (3.23–6.26)
**MVI**				
Presence vs. Absence	< 0.001	3.12 (2.66–3.66)	< 0.001	3.10 (2.53–3.80)

Variable	Tumor recurrence		OS	
	P	HR (95.0%CI)	*P*	HR (95.0% CI)
**Tumor capsule**				
Incomplete vs. Complete	< 0.001	1.48 (1.26–1.74)	< 0.001	2.16 (1.76–2.65)
**Edmondson–Steiner**				
III/IV vs. I/II	< 0.001	1.43 (1.19–1.72)	< 0.001	1.59 (1.25–2.03)
**Blood transfusion**				
Yes vs. No	0.01	1.34 (1.06–1.69)	0.22	1.21 (0.89–1.64)
**Surgical margin, cm**				
≤ 1 vs. >1	0.003	1.29 (1.09–1.52)	0.001	1.46 (1.17–1.81)
**Hepatectomy**				
Anatomical vs. Non–anatomical	0.98	1.00 (0.85–1.18)	0.65	1.05 (0.85–1.29)
**ACL**				
ACL vs. non–ACL	0.52	0.95 (0.80–1.12)	0.76	0.97 (0.78–1.20)

Supplementary Table S2: Clinicopathologic characteristics of patients treated with or without ACL with a score of ≥ 10

Variable	Primary cohort		P value	Validation cohort		*P* value
	Non–ACL	ACL		Non–ACL	ACL	
	(*n* = 71)	(*n* = 36)		(*n* = 24)	(*n* = 17)	
**Age, years**						
≤ 50	32 (45.1%)	26 (72.2%)	0.008**	15 (62.5%)	8 (47.1%)	0.33
> 50	39 (54.9%)	10 (27.8%)		9 (37.5%)	9 (52.9%)	
**Gender**						
Male	64 (90.1%)	31 (86.1%)	0.53	19 (79.2%)	14 (82.4%)	0.99*
Female	7 (9.9%)	5 (13.9%)		5 (20.8%)	3 (17.6%)	
**HBsAg**						
Positive	62 (87.3%)	30 (83.3%)	0.57	20 (83.3%)	12 (70.6%)	0.56*
Negative	9 (12.7%)	6 (16.7%)		4 (16.7%)	5 (29.4%)	
**HBeAg**						
Positive	20 (28.2%)	10 (27.8%)	0.97	6 (25.0%)	2 (11.8%)	0.51*
Negative	51 (71.8%)	26 (72.2%)		18 (75.0%)	15 (88.2%)	
**HBcAb**						
Positive	66 (93.0%)	34 (94.4%)	0.99*	24 (100.0%)	17 (100.0%)	0.99*
Negative	5 (7.0%)	2 (5.6%)		0 (0.0%)	0 (0.0%)	
**HCVAb**						
Positive	0 (0%)	1 (2.8%)	0.73*	1 (4.2%)	1 (5.9%)	0.99*
Negative	71 (100.0%)	35 (97.2%)		23 (95.8%)	16 (94.1%)	
**Cirrhosis**						
Yes	40 (56.3%)	16 (44.4%)	0.24	11 (45.8%)	8 (47.1%)	0.94
No	31 (43.7%)	20 (55.6%)		13 (54.2%)	9 (52.9%)	
**AFP, ng/mL**						
≤ 400	30 (42.3%)	19 (52.8%)	0.30	10 (41.7%)	11 (64.7%)	0.15
> 400	41 (57.7%)	17 (47.2%)		14 (58.3%)	6 (35.3%)	
**PT, second**						
≤ 12	34 (47.9%)	19 (52.8%)	0.63	11 (45.8%)	7 (41.2%)	0.77
> 12	37 (52.1%)	17 (47.2%)		13 (54.2%)	10 (58.8%)	

Variable	Primary cohort			Validation cohort		
	Non–ACL	ACL	P value	Non–ACL	ACL	*P* value
	(*n* = 71)	(*n* = 36)		(*n* = 24)	(*n* = 17)	
**PLT, ×10⁹/L**						
≤ 100	11 (15.5%)	4 (11.1%)	0.54	1 (4.2%)	4 (23.5%)	0.17*
> 100	60 (84.5%)	32 (88.9%)		23 (95.8%)	13 (76.5%)	
**ALB, g/L**						
≤ 40	33 (46.5%)	10 (27.8%)	0.06	7 (29.2%)	7 (41.2%)	0.42
> 40	38 (53.5%)	26 (72.2%)		17 (70.8%)	10 (58.8%)	
**ALT, U/L**						
≤ 40	36 (50.7%)	11 (30.6%)	0.05	14 (58.3%)	6 (35.3%)	0.15
> 40	35 (49.3%)	25 (69.4%)		10 (41.7%)	11 (64.7%)	
**TBIL, μmol/L**						
≤ 34	2 (2.8%)	0 (0.0%)	0.79*	0 (0.0%)	0 (0.0%)	0.99*
> 34	69 (97.2%)	36 (100.0%)		24 (100%)	17 (100%)	
**WBC, ×10⁹/L**						
≤ 4	10 (14.1%)	3 (8.3%)	0.58*	4 (16.7%)	5 (29.4%)	0.56*
> 4	61 (85.9%)	33 (91.7%)		20 (83.3%)	12 (70.6%)	
**Tumor number**						
Single	30 (42.3%)	22 (61.1%)	0.07	15 (62.5%)	15 (88.2%)	0.14*
Multiple	41 (57.7%)	14 (38.9%)		9 (37.5%)	2 (11.8%)	
**Tumor diameter, cm**						
≤ 3	0 (0.0%)	0 (0.0%)	0.99*	0 (0.0%)	0 (0.0%)	0.99*
3–5	0 (0.0%)	0 (0.0%)		0 (0.0%)	0 (0.0%)	
> 5	71 (100.0%)	36 (100.0%)		24 (100.0%)	17 (100.0%)	
**MVI**						
Presence	71 (100.0%)	36 (100.0%)	0.99*	24 (100.0%)	17 (100.0%)	0.99*
Absence	0 (0.0%)	0 (0.0%)		0 (0.0%)	0 (0.0%)	
**Tumor capsule**						
Complete	0 (0.0%)	0 (0.0%)	0.99*	0 (0.0%)	0 (0.0%)	0.99*
Incomplete	71 (100.0%)	36 (100.0%)		24 (100.0%)	17 (100.0%)	
**Edmondson–Steiner**						
I/II	8 (11.3%)	3 (8.3%)	0.89*	4 (16.7%)	1 (5.9%)	0.58*
III/IV	63 (88.7%)	33 (91.7%)		20 (83.3%)	16 (94.1%)	
**Blood transfusion**						
Yes	18 (25.4%)	7 (19.4%)	0.50	3 (12.5%)	6 (35.3%)	0.18*
No	53 (74.6%)	29 (80.6%)		21 (87.5%)	11 (64.7%)	
**Surgical margin, cm**						
≤ 1	70 (98.6%)	32 (88.9%)	0.08*	22 (91.8%)	17 (100.0%)	0.63*
> 1	1 (1.4%)	4 (11.1%)		2 (8.3%)	0 (0.0%)	
**Hepatectomy**						
Anatomical	48 (67.6%)	19 (52.8%)	0.13	17 (70.8%)	12 (70.6%)	0.99*
Non–anatomical	23 (32.4%)	17 (47.2%)		7 (29.2%)	5 (29.4%)	

*Continuity Correction test.
** The median ages of the non–ACL and ACL groups were 51.0 vs. 47.0 years ($p = 0.06$).

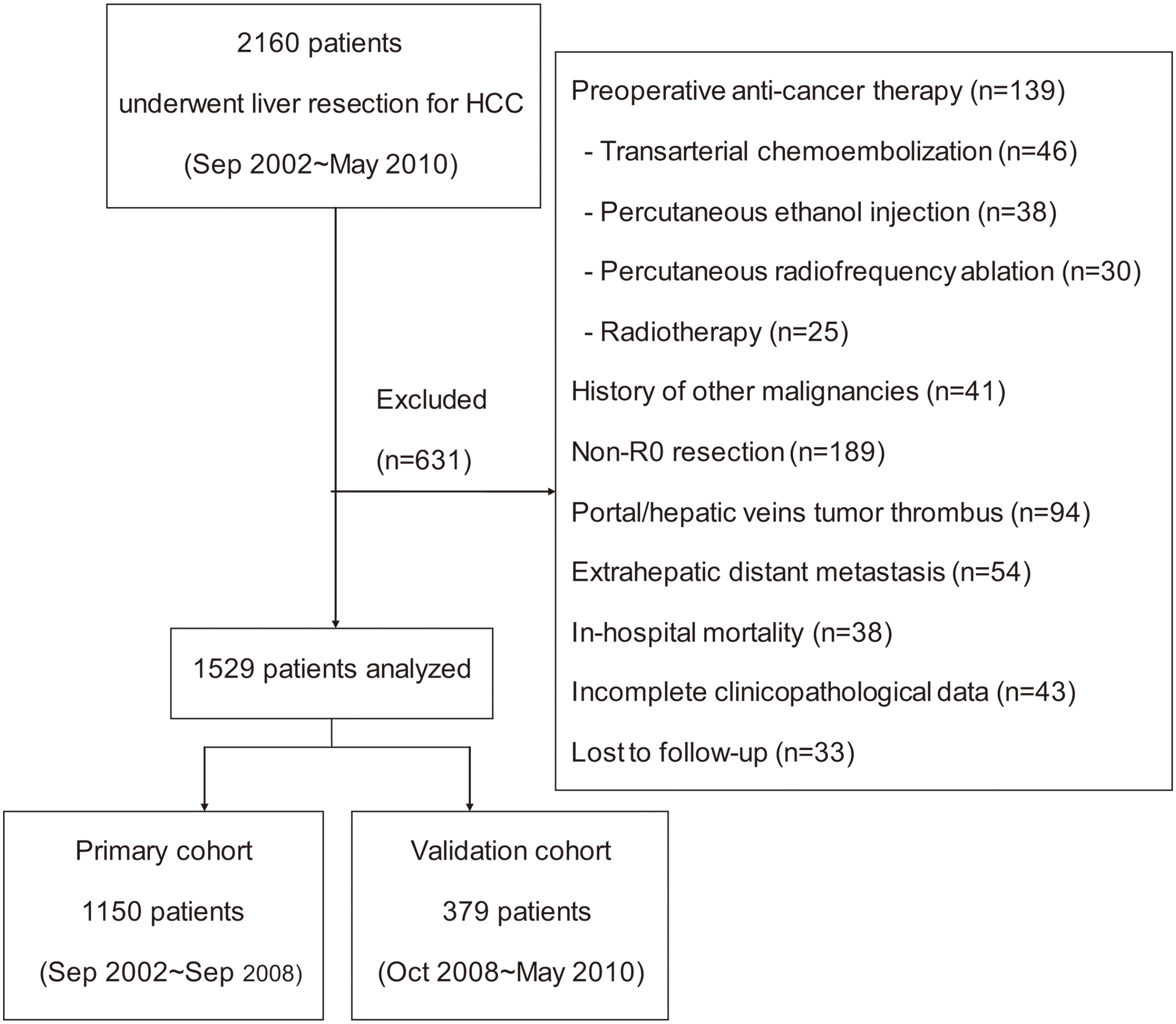

Supplementary Figure S1: Flow chart of the study.

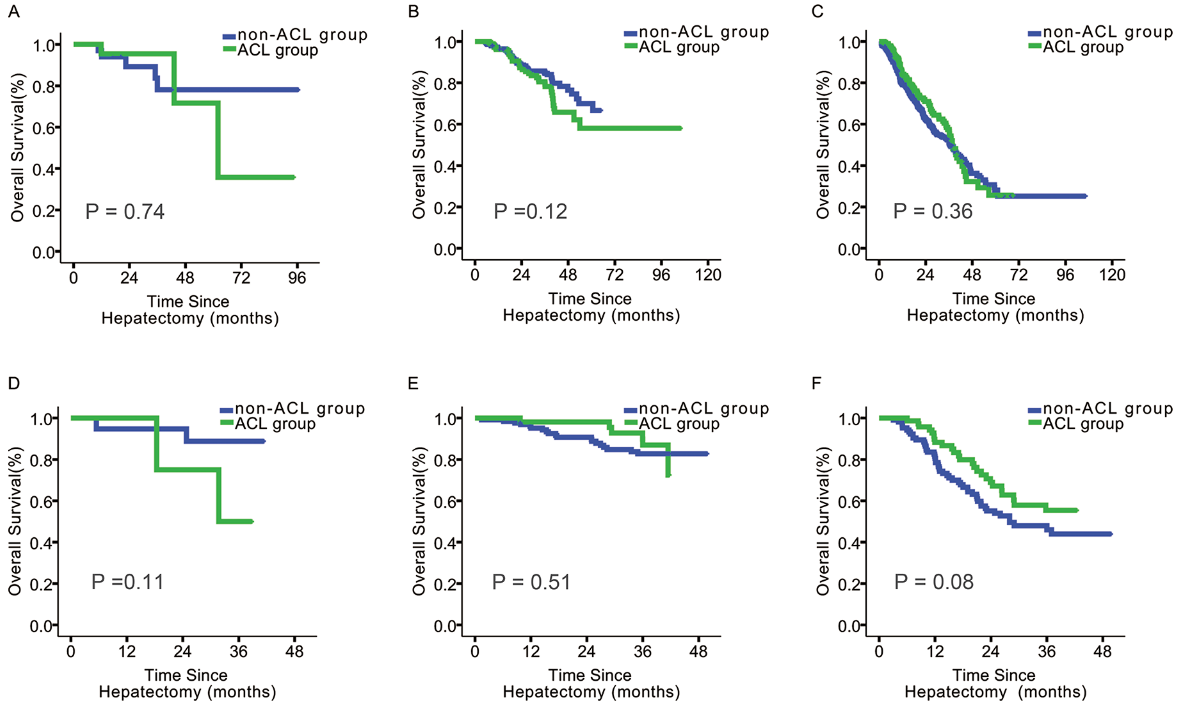

Supplementary Figure S2: Impact of ACL on OS in patients with HCCs at different BCLC stages. (**A, B, C**) OS of patients at stage 0, A and B in the primary cohort respectively; (p = 0.74, 0.12 and 0.36) (**D, E, F**) OS of patients at stage 0, A and B in the validation cohort respectively. (p = 0.11, 0.51 and 0.08).

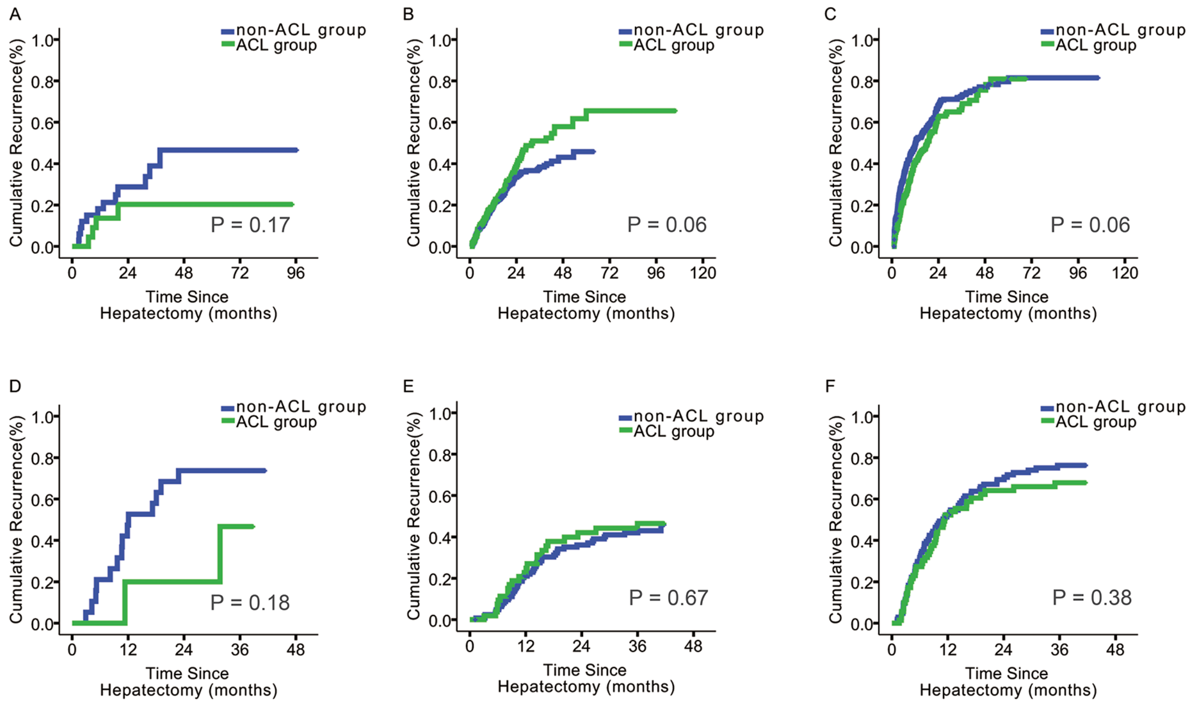

Supplementary Figure S3: Impact of ACL on tumor recurrence in patients with HCCs at different BCLC stages. (**A, B, C**) Tumor recurrence of patients at stage 0, A and B in the primary cohort respectively; (p = 0.17, 0.06 and 0.06) (**D, E, F**) Tumor recurrence of patients at stage 0, A and B in the validation cohort respectively. (p = 0.18, 0.67 and 0.38).

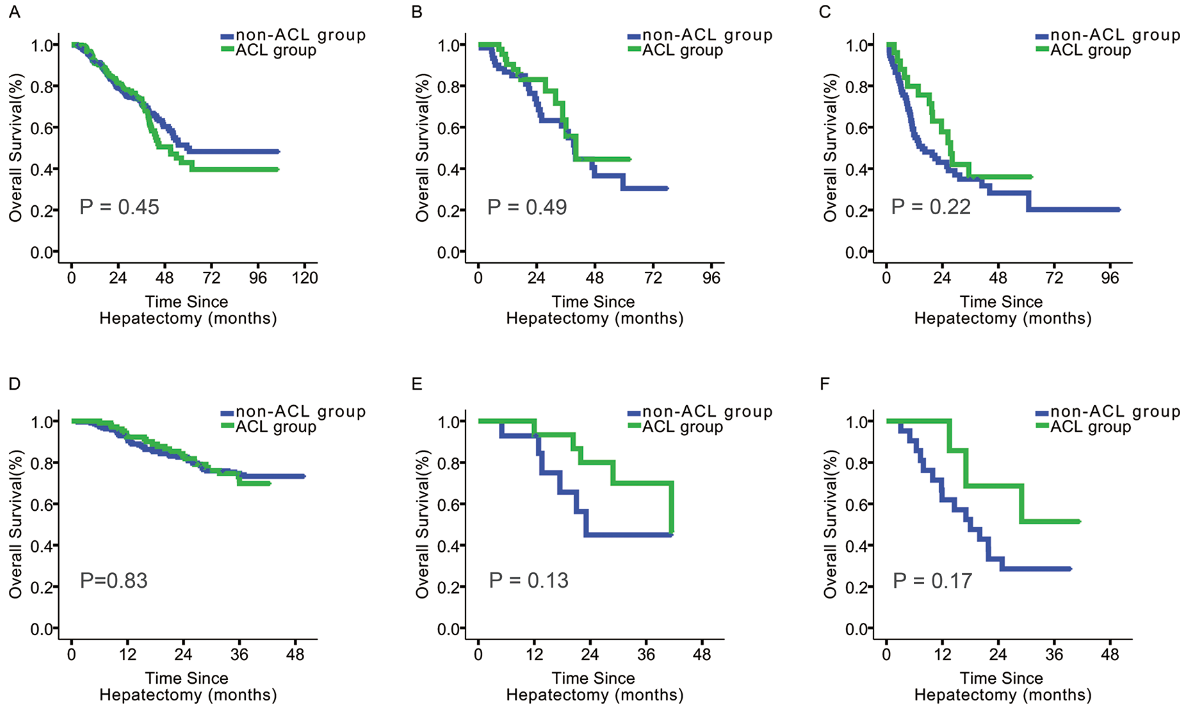

Supplementary Figure S4: Impact of ACL on OS in patients with HCCs at different 7th TNM stages. (**A, B, C**) OS of patients at stage I, II and III in the primary cohort respectively; (p = 0.45, 0.49 and 0.22) (**D, E, F**) OS of patients at stage I, II and III in the validation cohort respectively. (p = 0.83, 0.13 and 0.17).

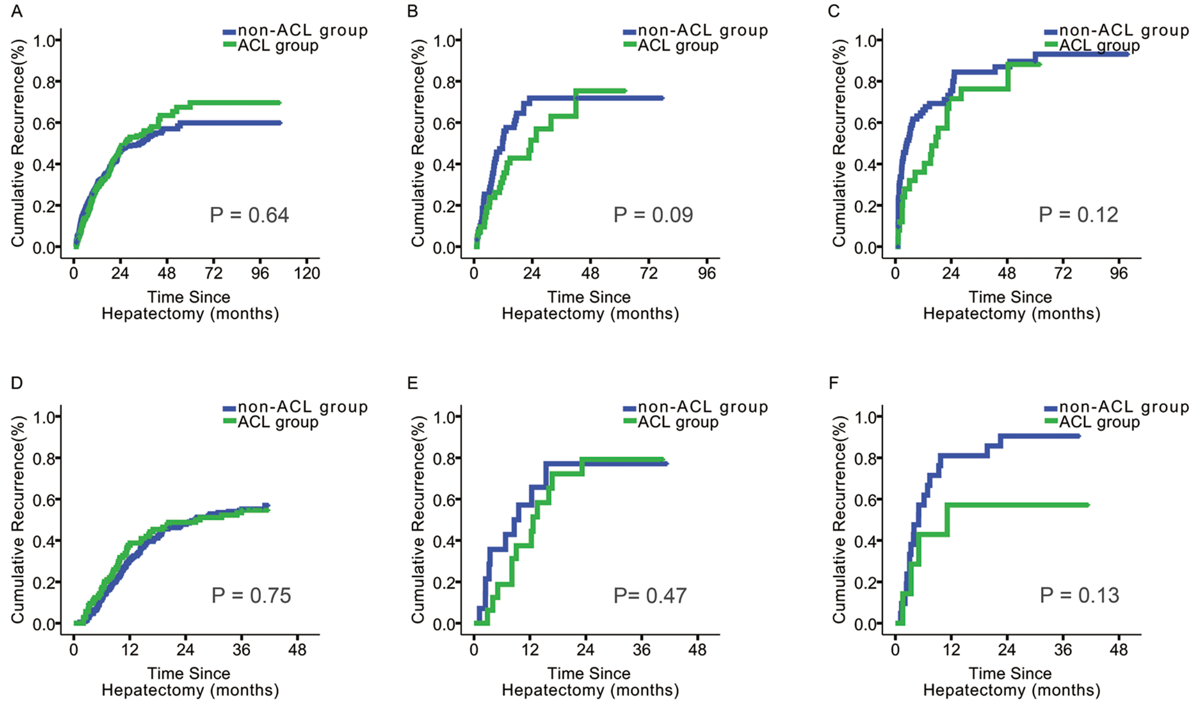

Supplementary Figure S5: Impact of ACL on tumor recurrence in patients with HCCs at different 7th TNM stages. (**A, B, C**) Tumor recurrence of patients at stage I, II and III in the primary cohort respectively; (p = 0.64, 0.09 and 0.12) (**D, E, F**) Tumor recurrence of patients at stage I, II and III in the validation cohort respectively. (p = 0.75, 0.47 and 0.13).
